# Genetic Testing for Rare Diseases: A Systematic Review of Ethical Aspects

**DOI:** 10.3389/fgene.2021.701988

**Published:** 2022-01-26

**Authors:** Judith Kruse, Regina Mueller, Ali A. Aghdassi, Markus M. Lerch, Sabine Salloch

**Affiliations:** ^1^ Institute of Ethics and History of Medicine, University Medicine Greifswald, Greifswald, Germany; ^2^ Institute of Ethics and History of Medicine, Medical Faculty, University Tübingen, Tübingen, Germany; ^3^ Department of Medicine A, University Medicine Greifswald, Greifswald, Germany; ^4^ LMU Munich University Hospital, Munich, Germany; ^5^ Institute of Ethics, History and Philosophy of Medicine, Hannover Medical School, Hannover, Germany

**Keywords:** genetic testing, rare diseases, orphan diseases, genetic councelling, ethics

## Abstract

Genetic testing is associated with many ethical challenges on the individual, organizational and macro level of health care systems. The provision of genetic testing for rare diseases in particular requires a full understanding of the complexity and multiplicity of related ethical aspects. This systematic review presents a detailed overview of ethical aspects relevant to genetic testing for rare diseases as discussed in the literature. The electronic databases Pubmed, Science Direct and Web of Science were searched, resulting in 55 relevant publications. From the latter, a total of 93 different ethical aspects were identified. These ethical aspects were structured into three main categories (process of testing, consequences of the test outcome and contextual challenges) and 20 subcategories highlighting the diversity and complexity of ethical aspects relevant to genetic testing for rare diseases. This review can serve as a starting point for the further in-depth investigation of particular ethical issues, the education of healthcare professionals regarding this matter and for informing international policy development on genetic testing for rare diseases.

## Introduction

Around 29 million people in the European Union (EU) (European Union, 2008), 30 million people in the Unites States of America (USA) (Global Genes, 2020) and around 400 million people worldwide are affected by one out of 5,000 to 8,000 different rare diseases (Global Genes, 2020). There is no uniform definition of rare diseases. A disease is considered as rare in the EU if it affects no more than 5 in 10,000 people (European Union, 2008), this definition will be adhered to in the following article. Half of the patients diagnosed with a rare disease are children and approximately 3% of newborns are affected by a rare disease (Global Genes, 2020; Eurordis, 2005). At least 80% of rare diseases have a genetic origin (Global Genes, 2020). This can mean either the involvement of one or several genes or chromosomal abnormalities. Often entire families or ethnic groups are affected due to the hereditary nature of the disease. However, rare diseases can be caused by *de novo* mutations affecting single individuals (Eurordis, 2005). Genetic and phenotypic variability add to the incomplete knowledge of rare diseases which complicates the process of diagnosis, leading to a diagnostic odyssey lasting an average of 8 years (Global Genes, 2020, Wright et al., 2018). This not only poses an immense strain and psychological distress on the patients and their families but also presents a serious challenge and burden to healthcare systems (Wright et al., 2018).

A precise molecular diagnosis is essential for the efficient handling of rare diseases in order to provide disease management and treatment options. In addition, it enables informed future family planning decisions and the formation of supportive networks of individuals and families affected by rare diseases (European Union, 2008; Wright et al., 2018). Early and precise diagnoses help to reduce further invasive and expensive testing and the psychological stress associated with an unknown diagnosis (Liu et al., 2019; Soden et al., 2012). A genetic diagnosis might not only be of interest for symptomatic individuals but can also be beneficial as a screening procedure in the identification of carriers and asymptomatic individuals and, thus, contributes to the secondary prevention of both benign and malignant diseases (Pulst, 2000).

Advances in genetic testing, especially next generation sequencing technologies (NGS), have positively impacted the likelihood of obtaining a genetic diagnosis in a timely manner (Wright et al., 2018; Liu et al., 2019, Soden et al., 2012). However, genetic testing still requires proper counseling prior to testing in order to obtain informed consent, and after the test when the results are delivered (Soden et al., 2012; Liu et al., 2019). If not properly understood by the patient, the disclosure of genetic test results might lead to adverse reactions, such as heightened anxiety and unnecessary precautionary measures (Committee on Bioethics, 2001). A positive test result in an individual might also provide genetic information about relatives who have not given their direct consent to this information. This can lead to communication challenges and brings the medical professional disclosing the information and the receiving patient into an uncomfortable position (Pulst, 2000; Ellis et al., 2001; Gross, 2002).

The rapid technological advancements in genetics and the lack of education in this field limit the ability of many nonspecialized physicians to partake in the much needed professional discussion of ethical issues in genetic testing (Pulst, 2000). The widespread lack of experience with rare diseases often only intensifies this problem. Issues of particular relevance for rare diseases include the ethical justification of testing for a condition that does not have treatment options available, which is the case for many rare diseases, or the seemingly ubiquitous risk of receiving a result of unknown or ambivalent significance and the necessary measures to follow (Boycott et al., 2013; Warman Chardon et al., 2015; Petrikin et al., 2015). Less obvious issues also need thorough ethical discussion, such as counseling for postmortem genetic testing, which is most relevant in instances of sudden unexpected deaths (Working Group commissioned by the Ontario Genetic Testing Advisory Committee, 2016; Tester and Ackermann, 2017). Additionally, accessibility of genetic testing itself can bear ethical challenges when routine care laboratories do not provide the tests and research settings remain the only option. Laboratories often lack any interest in providing genetic testing, especially for extremely rare diseases, since these tests have a low volume and the development and validation can be expensive (Ledbetter and Faucett, 2008).

This review is the first, to the best of the authors’ knowledge, to present a profound overview of all ethical aspects of genetic testing for rare diseases as published in the literature. In systematizing ethical problems related to this field this review can assist researches in the field of genetics as well as clinicians and counsellors in enhancing the moral sensibility for issues pertinent to their professional practice. For example, this review systematically gives a list of ethical issues occurring at the micro-level of patient-provider-contact and enables a further in-depth literature analysis of moral problems relevant for the individual reader. It furthermore provides a systematic basis for the ethical education of not only healthcare professionals but also patients, their families and other relevant stakeholders. This review provides a systematic background for further empirical and normative investigations of ethical aspects and is meant as a comprehensive aid to health policy making.

## Materials & Methods

### Aim

This article provides an overview of the full spectrum of ethical aspects in genetic testing for rare diseases based on a systematic review of the literature closely following the methodology used by Strech et al. (Strech et al., 2013). The reporting is in line with the PRISMA statement. The review does not aim to answer a specific normative-ethical question, but covers several ethical aspects related to genetic testing for rare diseases. The ethical aspects are qualitatively extracted from the publications and presented in a descriptive manner.

### Search Methods

**FIGURE 1:**
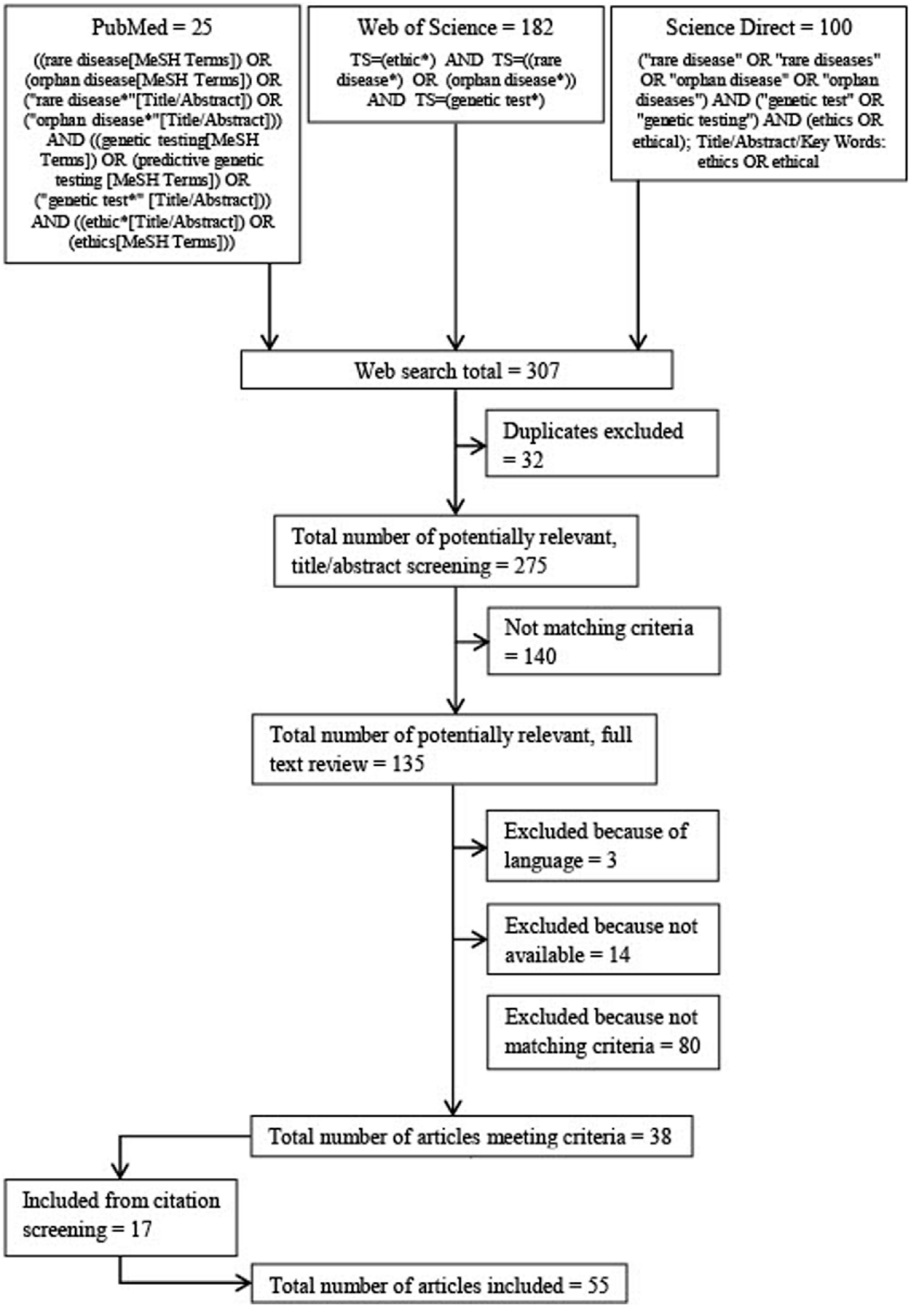
Flow diagram and search strings.

The electronic databases Pubmed, Science Direct and Web of Science were searched (see Figure 1 for search strings and flow diagram). The results of each search were downloaded and duplicates discarded. The database searches were conducted in June 2020. Language restrictions for the search were English and German.

### Eligibility Criteria

The only eligibility criterion for a publication to be included in the current systematic review was the description of an ethical aspect related to genetic testing for rare diseases.

“Rare diseases” are defined according to the EU as a disease affecting no more than 5 in 10,000 people (living in the EU) (European Union, 2008). Publications discussing rare diseases as a general topic were included as well as publications focusing on specific groups of rare diseases (e.g., neuromuscular disorders) or a single rare disease (e.g., Gaucher’s disease). Publications dealing exclusively with genetic screening, for example, newborn screening, were not included since this review focuses on predictive genetic testing rather than on population screening.

“Ethical aspects” were identified on the basis of the ethical theory of principlism, according to Beauchamp and Childress (Beauchamp & Childress, 2019). This approach defines four ethical principles: Respect for autonomy, non-maleficence, beneficence and justice. These four principles provide a general orientation and ought to be followed unless they conflict. If a conflict arises and not all principles can be followed, the conflicting principles need to be balanced in order to reach a solution. This act of balancing is always performed in the light of the specific situation.

“Genetic testing” is defined as an laboratory examination aimed at detecting or ruling out the presence of hereditary illnesses or predisposition to such conditions in a person by directly or indirectly analyzing their genetic heritage (e.g., genes, chromosomes, proteins) (European Union, 1997).

Up until the completion of this article, no definite set of criteria had been established on how to conduct a quality appraisal for reviews of ethical literature (Mertz, 2019). Consequently, no quality appraisal was conducted in the present review. An inadequate quality appraisal might withhold valuable features because the intention of this review is to display the full spectrum of ethical aspects.

**FIGURE 2:**
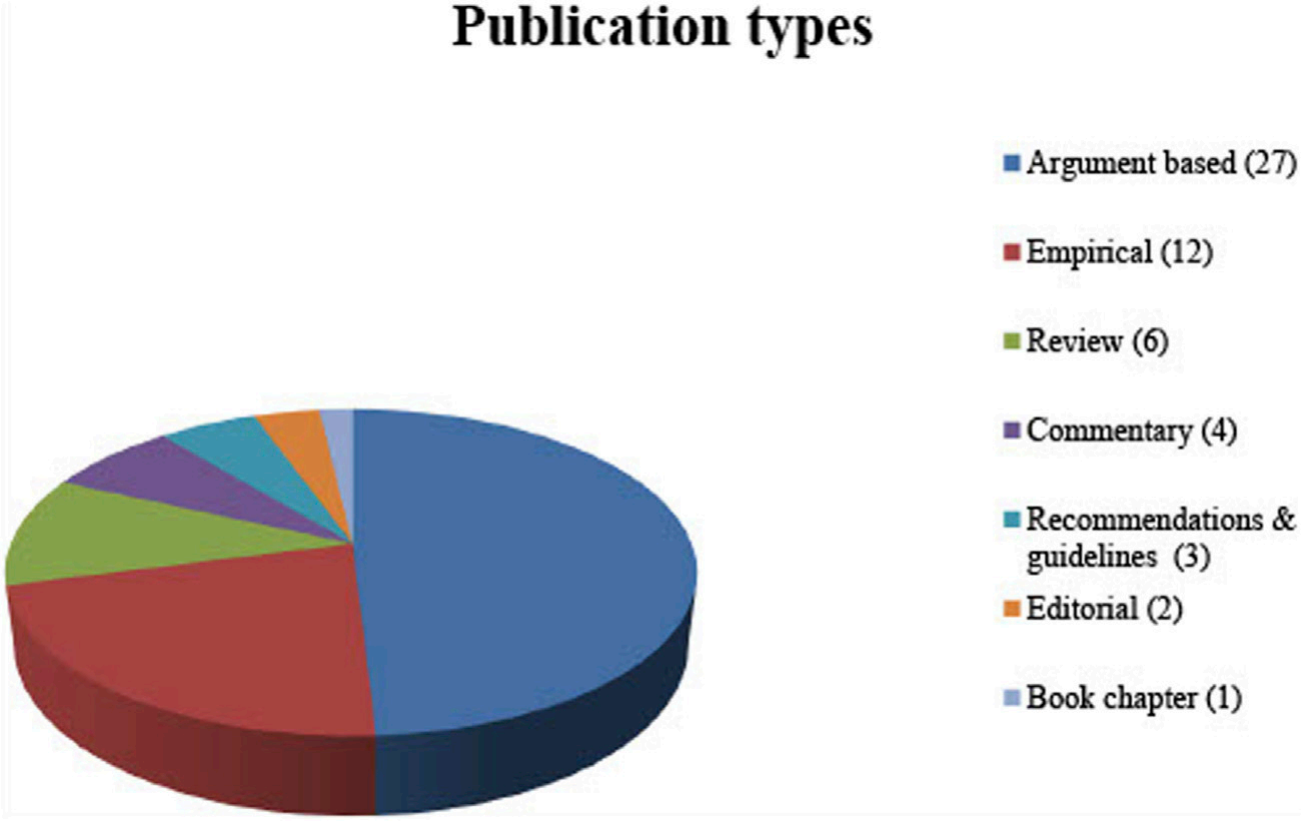
Publication types.

No restriction was applied to the type of publications included in this review. Therefore, not only original articles but also comments, editorials and book chapters were included. In order to display the full spectrum of ethical aspects relevant to the review question, not only argument-based but also empirical literature (when discussing ethical arguments) was included (See Figure 2 for publication types).

Similarly, no limit was established regarding the year of publication in order to include all ethical aspects mentioned and portray changes in the discussion over time.

### Study Selection

A title/abstract screening was performed on all publications retrieved from the databases searched. If publications appeared to meet the eligibility criteria, in a second step, the full text was analyzed. If a publication still met the criteria, i.e., addressed ethical aspects in the context of genetic testing for rare diseases, it was included in this review. After the inclusion of publications derived from the database search, an additional screening of all references and footnotes was conducted and supplementary publications were included based on the criteria described (see Figure 1 for search strings and flow diagram).

### Extraction and Synthesis of Ethical Aspects

The data were analyzed according to qualitative content analysis, as proposed by Mayring (Mayring, 2014), using the software MAXQDA12. The publications were screened for relevant text passages which, in a first step, were each assigned a descriptive code. These codes were then grouped if they described the same ethical aspect. These inductively derived codes were grouped in deductive categories and subcategories. They were regularly revised and altered to eliminate doubling or overlap to ensure the reliability of the coding system and the categories. Regular team meetings were held to discuss the coding procedure with all authors. The latter have academic backgrounds in medicine, applied ethics and philosophy.

## Results

The electronic database searches resulted in 307 publications published between 1988 and 2020. A total of 135 articles were identified by title abstract screening and the full texts were thoroughly examined based on the inclusion criteria. Eventually, 38 publications were included for systematic review. An additional 17 publications were identified by screening the citations. Fifty-four of these publications are written in English and one is written in German (see Table 1 for the publications included; see Figure 2 for the types of publication).

**TABLE 1:** Publications included.

1	Zhytnik L, Simm K, Salumets A, Peters M, Martson A, Maasalu K. Reproductive options for families at risk of osteogenesis imperfect: A review. Orphanet J. Rare. Dis. 2020; 15(1): 128.
2	Umbach N, Beißbarth T, Bleckmann A, Duttge G, Flatau L, König A, et al. Clinical application of genomic high-throughput data: Infrastructural, ethical, legal and psychosocial aspects. Eur Neuropsychopharmacol. 2020; 31: 1–15.
3	Marshall DA, MacDonald KV, Heidenreich S, Hartley T, Bernier FP, Gillespie MK, et al. The value of diagnostic testing for parents of children with rare genetic diseases. Genet Med. 2019; 21(12): 2798–2806.
4	Houdayer F, Putois O, Babonneau ML, Chaumet H, Joly L, Juif C, et al. Secondary findings from next generation sequencing: psychological and ethical issues. Family and patient perspectives. Eur J Med Genet. 2019; 62(10): 103711.
5	Bonnard A, Herson A, Gargiulo M, Durr A. Reverse pre-symptomatic testing for Huntington disease: double disclosure when 25% at-risk children reveal the genetic status to their parent. Eur J Hum Genet. 2019; 27(1): 22–27.
6	Normand EA, Alaimo JT, Van den Veyver IB. Exome and genome sequencing in reproductive medicine. Fertil Steril. 2018; 109(2): 213–220.
7	Hayeems RZ, Boycott KM. Genome-wide sequencing technologies: a primer for paediatricians. Paediatr Child Health. 2018; 23(3): 191–197.
8	Boardman FK, Young PJ, Warren O, Griffiths FE. The role of experiential knowledge within attitudes towards genetic carrier screening: A comparison of people with and without experience of spinal muscular atrophy. Health Expect. 2018; 21(1): 201–211.
9	Tester DJ, Ackerman MJ. Evaluating the survivor or the relatives of those who do not survive: the role of genetic testing. Cardiol Young. 2017; 27: 19–24.
10	Ravenscroft G, Davis MR, Lamont P, Forrest A, Laing NG. New era in genetics of early-onset muscle disease: Breakthroughs and challenges. Sem Cell Dev Biol. 2017; 64: 160–170.
11	Hayward J, Bishop M, Rafi I, Davison V. Genomics in routine clinical care: what does this mean for primary care? Br J Gen Pract. 2017; 67(655): 58–59.
12	Allen S, Young E, Bowns B. Noninvasive prenatal diagnosis for single gene disorders. Curr Opin Obstet Gynecol. 2017; 29(2): 73–79.
13	Afroze B, Brown N. Ethical issues in managing Lysosomal storage disorders in children in low and middle income countries. Pak J Med Sci. 2017; 33(4): 1036–1041.
14	Verhoef TI, Hill M, Drury S, Mason S, Jenkins L, Morris S, et al. Non-invasive prenatal diagnosis (NIPD) for single gene disorders: cost analysis of NIPD and invasive testing pathways. Prenat Diagn. 2016; 36(7): 636–642.
15	Smith LD, Willig LK, Kingsmore SF. Whole-exome sequencing and whole-genome sequencing in critically ill neonates suspected to have single-gene disorders. Cold Spring Harb Perspect Med. 2016; 6(2): a023168.
16	Working Group for the Use of Genome-Wide Sequencing for Undiagnosed Rare Genetic Diseases in Ontario. 2016
17	Warman Chardon J, Beaulieu C, Hartley T, Boycott KM, Dyment DA. Axons to exons: the molecular diagnosis of rare neurological diseases by next-generation sequencing. Curr Neurol Neurosci Rep. 2015; 15(9): 64.
18	Skirton H, Goldsmith L, Chitty LS. An easy test but a hard decision: ethical issues concerning non-invasive prenatal testing for autosomal recessive disorders. Eur J Hum Genet. 2015; 23(8): 1004–1009.
19	Petrikin JE, Willig LK, Smith LD, Kingsmore SF. Rapid whole genome sequencing and precision neonatology. Semin Perinatol. 2015; 39(8): 623–631.
20	Nguyen MT, Charlebois K. The clinical utility of whole-exome sequencing in the context of rare diseases - the changing tides of medical practice. Clin Genet. 2015; 88(4): 313–319.
21	Klein H-G, Rost I. Current methods in genetic analysis: an approach for genetics-based preventive medicine. Bundesgesundheitsblatt-Gesundheitsforschung-Gesundheitsschutz. 2015; 58(2): 113–120.
22	Sapp JC, Dong D, Stark C, Ivey LE, Hooker G, Biesecker LG, et al. Parental attitudes, values, and beliefs toward the return of results from exome sequencing in children. Clin Genet. 2014; 85(2): 120–126.
23	Might M, Wilsey M. The shifting model in clinical diagnostics: how next-generation sequencing and families are altering the way rare diseases are discovered, studied, and treated. Genet Med. 2014; 16(10): 736–737.
24	Lohmann K, Klein C. Next generation sequencing and the future of genetic diagnosis. Neurotherapeutics. 2014; 11(4): 699–707.
25	Lewis C, Hill M, Chitty LS. Non-invasive prenatal diagnosis for single gene disorders: experience of patients. Clin Genet. 2014; 85(4): 336–342.
26	Danielsson K, Mun LJ, Lordemann A, Mao J, Lin CH. Next-generation sequencing applied to rare diseases genomics. Expert Rev Mol Diagn. 2014; 14(4): 469–487.
27	Boardman FK. The expressivist objection to prenatal testing: the experiences of families living with genetic disease. Soc Sci Med. 2014; 107: 18–25.
28	Korf BR, Rehm HL. New approaches to molecular diagnosis. JAMA. 2013; 309(14): 1511–1521.
29	Kingsmore SF. Incidental swimming with millstones. Sci Transl Med. 2013; 5(194): 194ed10.
30	Boycott KM, Vanstone MR, Bulman DE, MacKenzie AE. Rare-disease genetics in the era of next-generation sequencing: discovery to translation. Nat Rev Genet. 2013; 14(10): 681–691.
31	Soden SE, Farrow EG, Saunders CJ, Lantos JD. Genomic medicine: evolving science, evolving ethics. Pers Med. 2012; 9(5): 523–528.
32	Makrythanasis P, Antonarakis SE. High-throughput sequencing and rare genetic diseases. Mol Syndromol. 2012; 3(5): 197–203.
33	Tester DJ, Ackerman MJ. Genetic testing for potentially lethal, highly treatable inherited cardiomyopathies/channelopathies in clinical practice. Circulation. 2011; 123(9): 1021–1037.
34	Kingsmore SF, Dinwiddie DL, Miller NA, Soden SE, Saunders CJ. Adopting orphans: comprehensive genetic testing of Mendelian diseases of childhood by next-generation sequencing. Expert Rev Mol Diagn. 2011; 11(8): 855–868.
35	Petrou M, Patrinos GP, Ansorge WJ. Genetic counseling and ethics in molecular diagnostics. In: Patrinos GP, Ansorge W (eds). Molecular Diagnostics. 2nd edn. (Academic Press, San Diego, 2010), pp. 537–548.
36	Fuentes J, Martín-Arribas MC. Bioethical issues in neuropsychiatric genetic disorders. Child Adolesc Psychiatr Clin N Am. 2007; 16(3): 649–661.
37	Lipinski SE, Lipinski MJ, Biesecker LG, Biesecker BB. Uncertainty and perceived personal control among parents of children with rare chromosome conditions: the role of genetic counseling. Am J Med Genet C Semin Med Genet. 2006; 142C(4): 232–240.
38	Dimichele D, Chuansumrit A, London AJ, Thompson AR, Cooper CG, Killian RM, et al. Ethical issues in haemophilia. Haemophilia. 2006; 12: 30–35.
39	Maddalena A, Bale S, Das S, Grody W, Richards S. Technical standards and guidelines: molecular genetic testing for ultra-rare disorders. Genet Med. 2005; 7(8): 571–583.
40	Kalfoglou AL, Scott J, Hudson K. PGD patients’ and providers’ attitudes to the use and regulation of preimplantation genetic diagnosis. Reprod BioMed Online. 2005; 11(4): 486–496.
41	Thomas SM. Society and ethics – the genetics of disease. Curr Opin Genet Dev. 2004; 14(3): 287–291.
42	Krajewski KM, Shy ME. Genetic testing in neuromuscular disease. Neurol Clin. 2004; 22(3): 481–508.
43	Delatycki BM, Powell LW, Allen KJ. Hereditary hemochromatosis genetic testing of at-risk children: What is the appropriate age? Genet Test. 2004; 8(2): 98–103.
44	Cox SM, Faucett WA, Chen B, Dequeker E, Boone DJ, McGovern MM, et al. International genetic testing. Genet Med. 2003; 5(3): 176–182.
45	Merz JF KA, Leonard DGB, Cho MK. Diagnostic testing fails the test. Nature. 2002: 577–579.
46	Gross ML. Ethics, policy, and rare genetic disorders: the case of Gaucher disease in Israel. Theor Med Bioeth. 2002; 23(2): 151–170.
47	Committee on Bioethics. Ethical issues with genetic testing in pediatrics. Pediatrics. 2001; 107: 1451–1455.
48	Ahmed S, Saleem M, Sultana N, Raashid Y, Waqar A, Anwar M et al. Prenatal diagnosis of beta-thalassaemia in Pakistan experience in a Muslim country. Prenat Diagn. 2000; 20: 378–383.
49	Pulst SM. Ethical issues in DNA testing. Muscle Nerve. 2000; 23(10): 1503–1507.
50	Thomas SM. Genomics: the implications for ethics and education. Br Med Bull. 1999; 55(2): 429–445.
51	Van der Riet AA, Van Hout BA, Rutten FF. Cost effectiveness of DNA diagnosis for four monogenic diseases. J Med Genet. 1998; 34: 741–745.
52	Gin BR. Genetic discrimination: Huntington’s disease and the Americans with Disabilities Act. Columbia L Rev. 1997; 97(5): 1406–1434.
53	Biesecker LG. Orphan tests. Camb Q Healthc Ethics. 1996; 5(2): 300–306.
54	Terrenoire G. Huntington’s Disease and the ethics of genetic prediction. J Med Ethics. 1992; 18: 79–85.
55	Morris M, Tyler A, Harper PS. Adoption and genetic prediction for Huntington’s disease. Lancet. 1988; 2(8619): 1069–1070.

A total of 918 relevant text passages were identified in the 55 publications included. These text passages were given descriptive codes, which were then pooled to a total of 93 different ethical aspects. These codes were grouped into three main categories: *Process of testing*, *consequences of the test outcome* and *contextual challenges*. A total of 20 subcategories were introduced within these main categories to structure the results further (see Table 2 for the coding system).

**TABLE 2:** Coding system.

Main category	Sub-category	Ethical aspect	Number of occurences	References
Process of testing			402	
	Availability		73	
		Collaboration of laboratories/specialists	10	(23) (24) (32) (34) (44) (53)
		Access to genetic testing	10	(1) (16) (20) (24) (28) (32) (39) (53)
		Research laboratories	43	(3) (20) (24) (30) (32) (33) (36) (39) (41) (42) (44) (49) (50) (53)
		Clinical laboratories	9	(16) (32) (33) (39) (44)
		Direct to consumer testing	1	(31)
	Consent		52	
		Informed consent process	39	(2) (9) (12) (15) (16) (18) (19) (20) (22) (26) (31) (33) (36) (38) (39) (41) (47) (50) (54)
		Consent with minors	3	(47) (49) (55)
		The right to know	4	(13) (19) (26) (54)
		The right not to know	5	(2) (8) (9) (21) (54)
		Tiered or dynamic forms of consent	1	(2)
	Genetic counseling		48	
		Difficulties of counseling	12	(1) (21) (32) (34) (37) (50) (54)
		Requirements	30	(1) (16) (19) (21) (28) (31) (33) (36) (37) (44)
		Retrospective counseling	3	(48) (51)
		Importance of genetic counseling	3	(9) (50)
	Timing of testing		73	
		Testing minors	7	(4) (25) (31) (47) (49)
		Relevance of timing	5	(6) (7) (13) (49) (55)
		Preimplantation genetic testing	34	(25) (27) (40) (47) (54)
		Testing for late-onset diseases	21	(19) (26) (31) (40) (41) (43) (47) (54) (55)
		Postmortem genetic testing	6	(9) (16) (17) (25) (27) (40) (47) (54)
	Interpretation of results		28	
		Interpretation	13	(9) (12) (19) (20) (26) (31) (32) (33) (50)
		Consequences of inaccurate interpretation	15	(17) (19) (26) (29) (33) (41) (47)
	Regulations and standards		56	
		Laboratory practice issues	17	(24) (44) (49) (53)
		Patient management issues	7	(44)
		Need for standards	13	(24) (28) (40) (44) (53)
		Patient/family as decision-maker	6	(40) (46) (54) (55)
		Protection from unethical practices	4	(20) (40) (53)
		Regulations creating barriers	9	(40)
	Physicians		22	
		Increased demands on physicians	17	(1) (7) (20) (24) (28) (29) (30) (31) (36) (37) (49)
		Family-professional collaboration	5	(23) (24)
	Reasons for testing		30	
		Clinical suspicion	7	(8) (22) (33)
		Desire to offer proper care	10	(1) (8) (14) (22) (31) (54)
		The need to know	9	(5) (22) (33)
		Reproductive choice	4	(1) (22) (47) (54)
	Other		20	
		Disclosure and access to the results	14	(2) (9) (26) (31) (33) (46) (54)
		Reasons not to test	6	(5)
Consequences of the test outcome			384	
	Diagnosis		78	
		End of diagnostic odyssey	15	(3) (6) (7) (10) (11) (17) (20) (23) (24) (29) (30) (34)
		Diagnostic certainty	31	(3) (7) (8) (9) (10) (11) (14) (15) (19) (20) (24) (25) (28) (31) (34) (38) (47) (49) (50)
		Prognosis	7	(7) (10) (15) (24) (31) (41) (51)
		Opportunities as a result of receiving diagnosis	12	(3) (7) (10) (14) (15) (25) (34) (37)
		Not receiving a molecular diagnosis	11	(17) (30) (33) (34)
		Positive effects of DNA diagnosis	1	(51)
		Social, personal and medical impacts of diagnosis	1	(3)
	Actionability of results		81	
		Access to disease-specific services	11	(15) (23) (24) (34) (54)
		Variants of unknown significance	5	(3) (9) (17) (19)
		Testing in the absence of therapeutic benefits	10	(6) (30) (32) (36) (49) (54)
		Prevention/alleviation of disease and suffering	25	(1) (2) (8) (10) (13) (15) (19) (25) (29) (34) (40) (47) (48) (50) (51)
		Disease management, therapy and interventions	30	(2) (3) (6) (10) (19) (24) (26) (29) (33) (34) (38) (41) (52)
	Incidental findings		53	
		Handling of incidental findings	14	(3) (2) (4) (7) (17) (19) (22) (24) (26) (31) (32) (36) (42) (49)
		Consequences of incidental findings	8	(4) (17) (26) (31) (42) (49)
		Consenting to receive incidental findings	8	(4) (17) (19) (26) (32) (49)
		Incidental findings in children	6	(7) (22) (26)
		Reporting recommendations	13	(7) (16) (17) (19) (26) (31) (32)
		Measures to decrease incidental findings	3	(10) (17) (31)
		Controversy: Proactively searching for unsolicited information	1	(7)
	Stigma and discrimination		41	
		Impact of stigma and discrimination	8	(1) (2) (8) (9) (28) (33) (38)
		Legislation to address discrimination	3	(28) (41)
		Discrimination by insurance companies	7	(21) (40) (41) (49) (50)
		Discrimination at the workplace	8	(21) (41) (50) (52)
		Adoption agencies/child welfare institutions	3	(52) (55)
		Other types of stigma and discrimination	11	(3) (31) (35) (40) (47) (52)
		Genetic testing used against people	1	(31)
	Family planning		59	
		Informed decision-making	17	(1) (2) (3) (6) (8) (10) (24) (27) (34) (42) (50) (52)
		Abortion/Termination	35	(1) (8) (10) (12) (13) (14) (18) (25) (27) (31) (35) (41) (46) (50) (51)
		Implications for future pregnancies	6	(1) (6) (8) (31)
		Social consequences of private reproductive decisions	1	(40)
	Involvement of relatives		41	
		Information about people not directly tested	4	(2) (9) (36) (49)
		Relevance of results to family members/others	17	(4) (5) (9) (11) (26) (28) (33) (36) (42) (46) (49) (50) (54)
		Disclosure to family	14	(2) (5) (9) (26) (28) (33) (41) (46) (49) (54)
		Paternal rights	6	(12) (18)
	Other		31	
		Uncertainty due to implications of the test result	8	(1) (36) (37) (46) (54)
		Awareness of disease	4	(8) (21) (34)
		Distress and adverse effects	19	(1) (3) (4) (5) (9) (22) (33) (34) (36) (47) (55)
Contextual challenges			132	
	Increased pressure to test		9	
		Coerced testing	4	(12) (25) (33) (49)
		Routinization of test usage	3	(12) (18) (27)
		Testing is optional	1	(6)
		Pressure to test in order to eradicate disease	1	(54)
	Economic aspects		40	
		Commercial interests restricting testing	9	(1) (14) (34) (40) (53)
		Dilemma if expensive test is used for information only	2	(14)
		Cost saving by genetic testing	16	(3) (6) (10) (13) (15) (19) (34) (40) (51)
		Genetic testing is expensive	7	(12) (14) (33) (40) (46) (53)
		Patents	3	(45)
		Large number of disorders is a cost challenge	1	(14)
		Difficulties to obtain funding	2	(15) (20)
	Data		24	
		Infrastructural challenges	7	(2) (26) (41) (49)
		Privacy concerns	11	(26) (33) (41) (46) (50) (54)
		Third parties using the data	2	(41) (49)
		Data sharing	4	(23) (26)
	Other		59	
		Rarity as a challenge	7	(12) (32) (34) (53)
		Difficulties in test developement	8	(20) (31) (53)
		Cultural differences	7	(1) (18) (48)
		Public understanding of genetic testing and rare diseases	10	(1) (27) (31) (40) (46)
		Effects on people living with a disability	6	(1) (18) (27) (40)
		Other ethical dilemmas	21	(1) (7) (8) (12) (15) (19) (20) (26) (31) (40) (41) (46) (53)

The following three main categories were established:

1) Process of testing: Ethical aspects concerning the procedure of genetic testing for rare diseases, the analysis of these tests and the delivery of the results to the patient and/or the family.
2) Consequences of the test outcome: Ethical aspects that result from the knowledge of the test result or the decisions made following the disclosure and patient reactions to the test result.
3) Contextual challenges: Ethical aspects that are associated with the circumstances and background of the tests, the diseases tested for and the test results.

**FIGURE 3:**
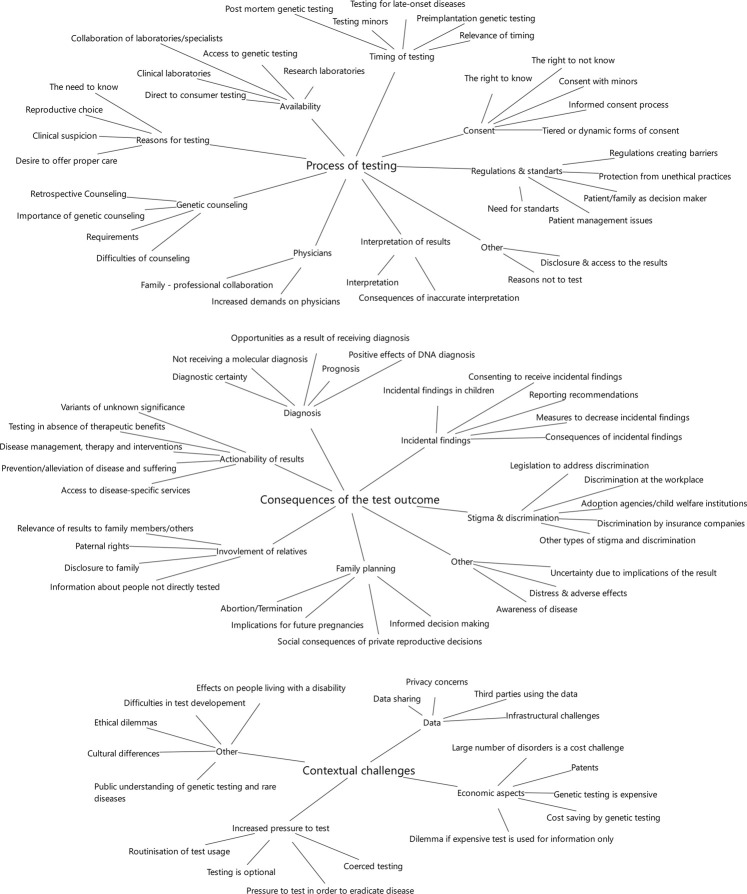
Categories.

### Process of Testing

The category *process of testing* encompasses 36 ethical aspects in nine subcategories (see Table 2 for the coding system and Figure 3 for the categories and subcategories). These aspects most often relate to practical issues which are prominent in routine clinician-patient-interactions such as obtaining informed consent or interpreting the test results. Accessibility is a broadly discussed ethical issue starting with the necessary referral to a testing facility which, however, presupposes the suspicion of a rare genetic disorder and knowledge of the testing opportunities:

“However, primary care physicians should be increasingly alerted to the new diagnostic options for patients with rare, unclassified conditions who may benefit from NGS-based genetic testing, and refer such patients to a center of rare diseases or similar tertiary care facility.”(Lohmann and Klein, 2014)

A challenge is seen in finding local or even national laboratories providing adequate testing for rare diseases and especially ultra-rare diseases:

“In contrast, for rare genetic conditions, testing may be available from very few laboratories, necessitating specimen and patient referrals across national boundaries.” (Cox et al., 2003)

Additionally, tests might be offered in research settings only, which further complicates the process of counselling:

“[…] some tests exist in a gray area between the research and clinical worlds, either temporarily because they are in transition, or permanently because there is no market for the test.” (Biesecker, 1996)

An inevitable component of genetic counselling is obtaining informed consent for the testing procedure and the disclosure of the results. In view of the rapidly evolving field of genetic technologies and the very specific characters of the genetic disorders the counsellor is confronted with major communicative challenges:

“[…] individualization may need to be integrated more into the informed consent process in order to accommodate individual preferences, allowing individuals the autonomy to opt in or out of receiving more extensive information regarding results.” (Danielsson et al., 2014)

The subcategory “timing of testing” is very prominent in the ethical literature included in the review. Several different time frames are discussed, including preimplantation of genetic testing, genetic testing during pregnancy, genetic testing in minors or testing for late onset diseases. These are especially sensitive topics about which patients might have preconceived notions which need to be respectfully addressed during counselling. Only post mortem genetic testing has not been prominently discussed even though coming with difficulties in obtaining consent and respect for the deceased.

### Consequences of the Test Outcome

The category c*onsequences of the test outcome* consists of 37 ethical aspects in seven subcategories. It deals with aspects resulting directly from the disclosure of the outcome (see Table 2 for the coding system and Figure 3 for the categories and subcategories). This category is relevant for a wide range of health care professionals as it focuses on potentially far-reaching clinical consequences and has major implications for provider-patient-communication.

Some of the consequences of the test outcome were judged as *benefits*; others are rather negatively connoted. The positive aspects include the knowledge about the condition and its therapy. The diagnosis ideally leads to accessing treatment options but is sometimes viewed as insufficient if no direct treatment is available. This situation is challenging and should be anticipated in research and clinical practice:

“Even if we know the genes involved and the loci associated with the condition, this does not mean that we have an immediate specific line of treatment. This mismatch between risk information and the possibility of effective treatment is one of the sources of ethical, legal, and social conflicts with which researchers and clinicians should be familiar.” (Fuentes and Martín-Arribas, 2007)

But also less obvious options such as becoming part of a patient self-help group and connecting *via* social media with individuals and families with similar diagnoses might be the consequence of a molecular diagnosis:

“A diagnosis also provides the family an opportunity to connect with disease-specific support groups so that they might meet others affected by the same rare disorder and exchange information about useful therapies and educational strategies.” (Kingsmore et al., 2011)

This community aspect is particularly relevant in situations where no conventional treatment options are available. In this case interprofessional care plays a prominent role as an increased coordination of care among providers will become necessary to provide best supportive care.

Further ethical issues involve the relevance of the test result for people who are not directly tested. By disclosure of their results the family members learn of very private information about themselves (and further relatives) which they did not consent to but if not disclosed might have an interest to know. This ambiguous situation needs to be extensively addressed and prepared for during counselling:

“Ethical challenges are generated when information produced by the results may affect third parties, including family members not directly involved in the process.” (Fuentes and Martín-Arribas, 2007)

### Contextual Challenges

The category c*ontextual challenges* includes 21 ethical aspects in four subcategories (see Table 2 for the coding system and Figure 3 for the categories and subcategories). These aspects do not refer directly to the procedure or result of the test but rather address the general circumstances of genetic testing for rare diseases, including societal aspects. In this, the contextual aspects typically lie out of the direct sphere of influence of clinicians and genetic counsellors but touch more generally on health care structures, policies and frameworks.

For example, it is argued, that it is inevitable to be aware of contextual challenges in order to integrate patients’ experiences and expectations into appropriate support and care. Optimal research and care, however, are often hindered by the contingent character of rare diseases:

“The application of evidence-based medicine in the field of genetic testing often remains questionable, because rare diseases imply a difficulty to meet the criteria required in terms of sample size for clinical trials or sound genetic research.” (Fuentes and Martín-Arribas, 2007)

Further ethical issues extend to economic aspects influencing the provision and development of genetic tests for rare diseases. Depending of the point of view genetic testing for rare diseases can be very cost effective as in cutting back the diagnostic odyssey of many years but are, as a single test, often still expensive. Here societal dialogue is necessary to examine the costs and benefits not only for the individual patient but also the society as a whole. On a macro level the financing of healthcare systems prominently intersects with the provision of care:

“Patients with rare diseases traditionally experience a prolonged and expensive diagnostic odyssey culminating in a delayed diagnosis or, frequently, no diagnosis at all. […] this diagnostic odyssey is a financial burden to the health-care system, costing more than US $10,000 per patient.” (Marshall et al., 2019)

An aspect commonly brought up in the economic discussion is the question of testing without consequences, when the test is used for information purposes only. This leads to delicate situations in clinical care, for example, when such a testing is performed during pregnancy:

“The expected increased uptake of NIPD [non-invasive prenatal diagnosis] […] highlight the ethical issues associated with using NIPD for information only and the appropriateness of directing resources to a test that would not change pregnancy management […]. Decisions about how NIPD is offered will need to take this concern into consideration, keeping in mind […] the clinical and psychological benefits afforded by NIPD which include early the possibility of reassurance or provision of information for planning and preparation of the birth of an affected child, as well as the potential of access to surgical termination of pregnancy.” (Verhoef et al., 2016)

The contextual challenges of genetic testing for rare diseases eventually include different ethical fields such as the methodology of clinical trials, issues of distributional justice and dealing with diagnoses without appropriate treatment options.

**FIGURE 4:**
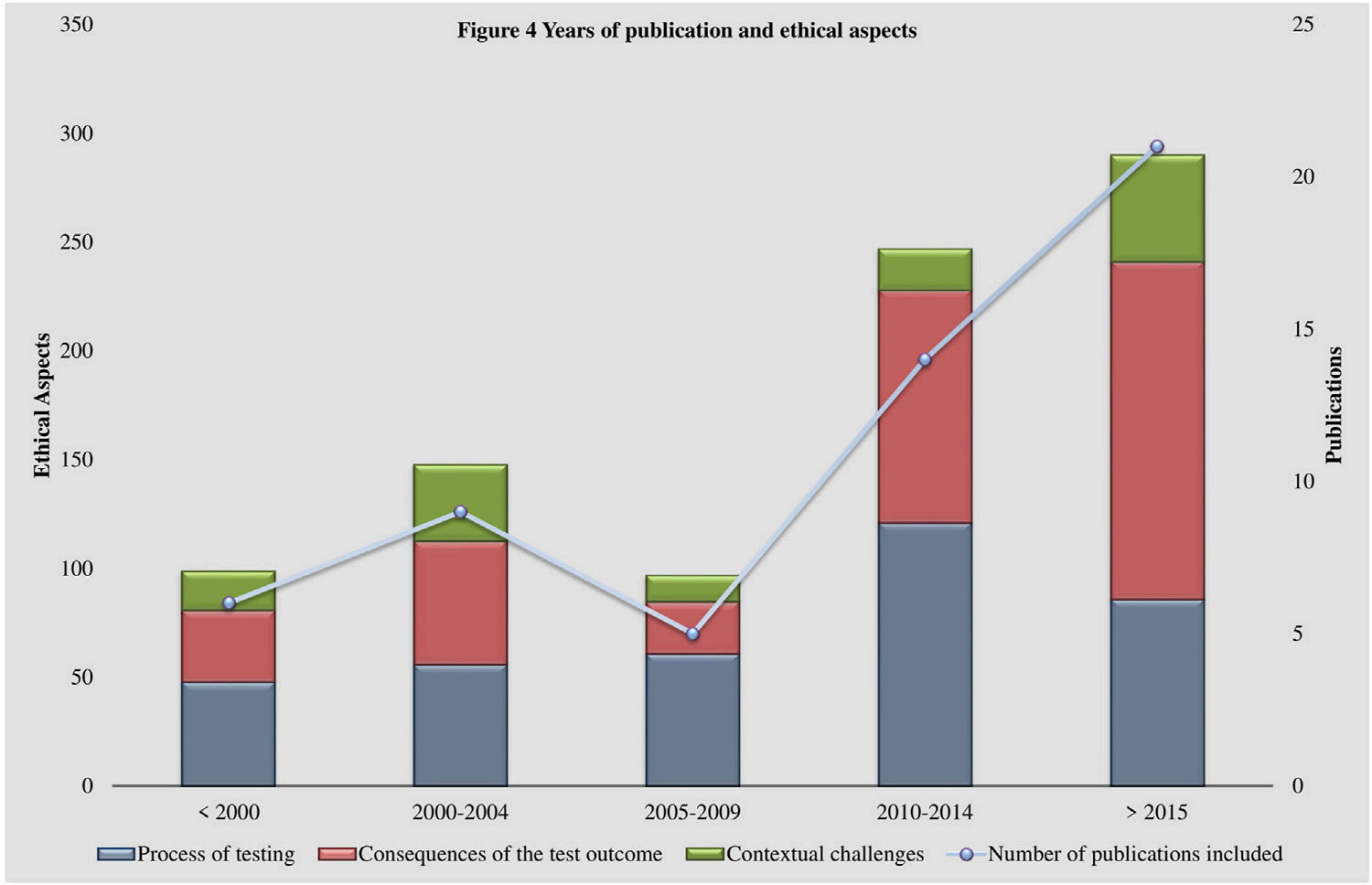
Years of publication and ethical aspects.

Some shifts in the perspectives of the discussion of ethical aspects over the past 30 years are visible within the coding system (See Figure 4 Years of publication and ethical aspects). Literature published in the 1990s and early 2000s focuses more on practical aspects, such as regulation and standards concerning laboratory practices (Terrenoire, 1992; Biesecker, 1996; Pulst, 2000; Cox et al., 2003). At that time, the establishment of guidelines was essential to address appropriately the rapidly developing technologies. Additionally, uncertainties about the possible impacts of the fast rising usage of genetic testing technologies were widely prevalent. Possible scenarios of discrimination resulting from the disclosure of a genetic test result were brought up (Thomas, 1999; Gin, 1997; Committee on Bioethics, 2001; Thomas, 2004) and the handling and security of genetic data was reflected (Terrenoire, 1992; Thomas, 1999; Pulst, 2000; Gross, 2002; Thomas, 2004). Interestingly, data management and security remained important issues to this day but have left the center of the debate (Cox et al., 2003; Umbach et al., 2020). The perspectives of more recent publications, in alignment with advancements in genetic testing such as whole genome sequencing, have shifted to more specific questions, such as the handling of incidental findings (Marshall et al., 2019; Houdayer et al., 2019; Umbach et al., 2020). The obtainment of incidental findings is closely related to the establishment of new technologies (Umbach et al., 2020). This raises questions regarding their utility, consent and, thereby, aggravates the appropriate delivery of results (Umbach et al., 2020). Similarly, the indispensable role of the physician in interpreting and disclosing the results (Nguyen and Charlebois, 2015; Hayeems and Boycott, 2018; Zhytnik et al., 2020) and their relationship to the patient (Lohmann and Klein, 2014; Might and Wilsey, 2014) have gained significance in the literature.

## Discussion

The appropriate handling of ethical issues is a requisite for adequate care in patients undergoing genetic testing for rare diseases. A variety of publications, using different methods and focal points, dealing with a multiplicity of ethical aspects were identified in this review. Positive, negative and ambiguous aspects were found which portray the challenges with which individuals, families, professionals, healthcare systems and society are faced. A thorough understanding of their diversity and complexity is a prerequisite for attending to ethical aspects systematically and transparently. In the light of the diversity of ethical issues one aspect from each major coding category will be discussed exemplarily in the following while also highlighting the intersection with other relevant ethical aspects.

### Process of Testing

An aspect which was discussed most controversially in the literature published in the recent 10 years and which is of particular relevance to rare diseases, in contrast to more common diseases, is the availability and accessibility of genetic testing. Genetic testing specifically in the research setting was intensively discussed by various authors (Biesecker, 1996; Fuentes and Martín-Arribas, 2007; Marshall et al., 2019). Many patients with a suspected rare disease find themselves in a situation where their only access to a genetic test is to be found in a study context, since many genetic tests for rare diseases are unattractive to clinical laboratories for their low profitability (Biesecker, 1996; Thomas, 2004; Marshall et al., 2019).

Missing opportunities to undergo a genetic test outside the research setting might compromise the key ethical requirement of voluntary participation in research. In addition, other regulations apply to research laboratories than to clinical laboratories (Biesecker, 1996; Pulst, 2000; Might and Wilsey, 2014). Standards for test validity and reliability differ from those in clinical laboratories, further complicating patient communication and the utilization of results (Biesecker, 1996; Danielsson et al., 2014). Also standards for the return of results vary, patients often receive the results only after completion of the study, while appropriate counseling is not always guaranteed (Krajewski and Shy, 2004; Danielsson et al., 2014; Might and Wilsey, 2014). This leaves the referring physician with a particularly difficult task of navigating this very specific setting with their patient and the family.

The intersection between clinical care and study context is only superficially addressed in national and international policies. One example is “The Council of Europe Convention on Human Rights and Biomedicine” which also covers genetic testing. Article 12 of the Convention limits the usage of predictive genetic testing to that it “[…] may be performed only for health purposes or for scientific research linked to health purposes […]” (European Union, 1997). Thereby, genetic testing in research setting is technically covered by the convention as a minimum standard but no elaboration is offered concerning this specific situation. Furthermore, as of June 2020 this convention has only been ratified by 29 members of the European Council (Tester and Ackermann, 2017).

### Consequences of the Test Outcome

One frequently discussed outcome of genetic testing is the possibility of facing stigma and discrimination (Korf and Rehm, 2013; Dimichele et al., 2006; Tester and Ackermann, 2017). According to the definition of E. Goffman a stigma describes a distinctive feature of a person which is linked to a negative stereotype and is considered “deeply discrediting” by societal standards (Goffman, 1963). Stigma and discrimination might come in a multitude of forms such as regulatory issues regarding insurance or employment or social issues such as exclusion from social activities.(Williams et al., 2010; Tester and Ackerman, 2011). Stigmatization can apply not only to people affected by a diagnosis, but also those with a positive carrier status. In specific cultural contexts this stigmatization could cause gender specific discrimination and reproductive restrictions for some women (Zhytnik et al., 2020). In the United States, as an example, in 2009 the Genetic Information Nondiscrimination Act (GINA) was signed which protects patients from being denied employment and health insurance based on their genetic test results (Tester and Ackerman, 2011). This menace of stigmatization and discrimination raises the questions, who should have access to genetic information and how to best ensure confidentiality and data protection while still utilizing the test results to their full potential (Umbach et al., 2020).

One aspect which was not represented in this review is that stigma and genetic discrimination are not universal experiences for everyone diagnosed with a genetic disease but highly individual experiences which might even have differing outcomes (Williams et al., 2010). A genetic cause is not automatically connected to a sense of being stigmatized (Sankar et al., 2006). A hereditary disease might also mean growing up among people with the same condition and impairments. Therefore, a genetic diagnosis might become a positive feature of identity, as for example being the basis for family cohesiveness, or as a link to one’s ancestry (Sankar et al., 2006).

Additionally, a diagnosis might also imply a connection to other affected individuals outside the family which can be accessed locally in the form of support groups or globally in online supportive networks (Kingsmore et al., 2011; Petrikin et al., 2015). Useful information about the condition, potential treatment options or supportive services can be exchanged alleviating the commonly sparse information available about genetic conditions. Even other forms of support such as psychological or spiritual assistance will be easier to access once the condition and prognosis are clear (Petrikin et al., 2015). This can be especially beneficial in situations where a diagnosis does not come along with curative therapeutic options.

### Contextual Challenges

Unexpectedly, not one of the publications included has economics aspects as its main focus. Of the publications included, 17 discuss an economic aspect but only four publications include two or more aspects. The reason for this neglect of economic aspects remains rather unclear. The EU and several individual states have legislations in place to foster the research on medicinal products for rare diseases as well as the provision of those (European Union, 2008). These legislations do not include medical devices such as genetic tests (European Union, 2008). Therefore, laboratories are not provided with an economic incentive for developing such tests (Hayeems and Boycott, 2018). Some of the authors voiced concern about the exclusivity of patents on genes and how they contribute significantly to the high prices of genetic tests for rare diseases (Nguyen and Charlebois, 2015). Thereby, the tests become even less attractive to clinical laboratories, perpetuating the testing in research laboratories discussed later (Nguyen and Charlebois, 2015). Patented genes offer the owner a monopoly not only of the initial diagnostic test but every diagnostic method using the same DNA sequence (Might and Wilsey, 2014). Thereby, the already difficult access to testing is at risk of being further compromised on behalf of patent interests.

### Limitations

This review aims to offer an empirical analysis portraying the diversity and complexity of ethical aspects relevant to genetic testing for rare diseases. The purpose of this review was not to quantify how often certain ethical aspects have been mentioned in the literature. Furthermore, it is important to understand that the frequency with which an ethical aspect occurs does not necessarily correlate to its relevance or importance. The ethical aspects displayed in this review are limited to the publications found via the three databases accessed (Pubmed, Science Direct, Web of Science). Prior to the final search, an exploratory search of several databases was conducted and the three databases subsequently accessed were identified as delivering the highest number of relevant results. Additionally, the neglect of literature written in languages other than English or German limits this review.

No quality appraisal for the included literature was performed due to the lack of quality assessment tools for systematic reviews of ethical literature (Mertz, 2019). Therefore, all publications fitting the inclusion criteria were included and it is up to the readership to critically judge the quality of the ethical aspects presented.

## Conclusion

A lack of knowledge and comprehension of the fast-paced developments of genetic testing among professionals poses an obstacle to accessing comprehensive testing (Nguyen and Charlebois, 2015). Many physicians find themselves insufficiently equipped for processing the amount of information that accompanies a genetic testing result for a rare disease and feel overwhelmed when navigating the complex ethical aspects associated (Pulst, 2000; Sankar et al., 2006; Soden et al., 2012). This is only intensified by the diversity of rare diseases themselves and the widespread lack of knowledge and awareness about them, and needs to be addressed (Soden et al., 2012).

This review found that not many physicians find themselves in a position where they feel knowledgeable enough to order and conduct genetic testing, especially for rare diseases (Soden et al., 2012). An effective cooperation with genetic counsellors forms the basis to solving this issue. These counsellors are specially trained non-physician experts in genetics who should be an integral part of every inter-professional genetics team (Soden et al., 2012; Boycott et al., 2013; Fuentes and Martín-Arribas, 2007).

However, this should, on the other hand, not deviate from the much needed extension of the education of physicians and other healthcare professionals to deliberately cover the advantages and disadvantages of genetic testing in the context of rare diseases, including not only medical subjects but also the ethical and legal issues presented in this review (Gin, 1997; Soden et al., 2012; Umbach et al., 2020).

## Data Availability

The original contributions presented in the study are included in the article/Supplementary Material, further inquiries can be directed to the corresponding author.
